# Molecular cloning and characterization of a novel pyrethroid-hydrolyzing esterase originating from the Metagenome

**DOI:** 10.1186/1475-2859-7-38

**Published:** 2008-12-30

**Authors:** Gang Li, Kui Wang, Yu Huan Liu

**Affiliations:** 1 State Key Laboratory of Biocontrol, School of Life sciences, Sun Yat-sen University, Guangzhou, 510275, PR China

## Abstract

**Background:**

Pyrethroids and pyrethrins are widely used insecticides. Extensive applications not only result in pest resistance to these insecticides, but also may lead to environmental issues and human exposure. Numerous studies have shown that very high exposure to pyrethroids might cause potential problems to man and aquatic organisms. Therefore, it is important to develop a rapid and efficient disposal process to eliminate or minimize contamination of surface water, groundwater and agricultural products by pyrethroid insecticides. Bioremediation is considered to be a reliable and cost-effective technique for pesticides abatement and a major factor determining the fate of pyrethroid pesticides in the environment, and suitable esterase is expected to be useful for potential application for detoxification of pyrethroid residues. Soil is a complex environment considered as one of the main reservoirs of microbial diversity on the planet. However, most of the microorganisms in nature are inaccessible as they are uncultivable in the laboratory. Metagenomic approaches provide a powerful tool for accessing novel valuable genetic resources (novel enzymes) and developing various biotechnological applications.

**Results:**

The pyrethroid pesticides residues on foods and the environmental contamination are a public safety concern. Pretreatment with pyrethroid-hydrolyzing esterase has the potential to alleviate the conditions. To this end, a pyrethroid-hydrolyzing esterase gene was successfully cloned using metagenomic DNA combined with activity-based functional screening from soil, sequence analysis of the DNA responsible for the *pye3 *gene revealed an open reading frame of 819 bp encoding for a protein of 272 amino acid residues. Extensive multiple sequence alignments of the deduced amino acid of Pye3 with the most homologous carboxylesterases revealed moderate identity (45–49%). The recombinant Pye3 was heterologously expressed in *E. coli *BL21(DE3), purified and characterized. The molecular mass of the native enzyme was approximately 31 kDa as determined by gel filtration. The results of sodium dodecyl sulfate-polyacrylamide gel electrophoresis and the deduced amino acid sequence of the Pye3 indicated molecular mass of 31 kDa and 31.5 kDa, respectively, suggesting that the Pye3 is a monomer. The purified Pye3 not only degraded all pyrethroid pesticides tested, but also hydrolyzed ρ-nitrophenyl esters of medium-short chain fatty acids, indicating that the Pye3 is an esterase with broader specificity. The K_m _values for *trans*-Permethrin and *cis*-permethrin are 0.10 μM and 0.18 μM, respectively, and these catalytic properties were superior to carboxylesterases from resistant insects and mammals. The catalytic activity of the Pye3 was strongly inhibited by Hg^2+^, Ag^+^, ρ-chloromercuribenzoate, whereas less pronounced effect was observed in the presence of divalent cations, the chelating agent EDTA and phenanthroline.

**Conclusion:**

A novel pyrethroid-hydrolyzing esterase gene was successfully cloned using metagenomic DNA combined with activity-based functional screening from soil, the broader substrate specificities and higher activity of the pyrethroid-hydrolyzing esterase (Pye3) make it an ideal candidate for in situ for detoxification of pyrethroids where they cause environmental contamination problems. Consequently, metagenomic DNA clone library offers possibilities to discover novel bio-molecules through the expression of genes from uncultivated bacteria.

## Background

Pyrethroids and pyrethrins are widely used insecticides. Natural pyrethrins are compounds with insecticidal activity extracted from chrysanthemum flowers. Pyrethroids are synthetic compounds similar in structure to pyrethrins, which have been modified over the years to enhance their insecticidal activity and/or their persistence in the environment [1]

Pyrethroids are likely to become more widely used as organophosphate insecticides including diazinon and chlorpyrifos are phased out due to the concerns regarding their safety [2]. Extensive applications not only result in pest resistance to these insecticides, but also may lead to environmental issues and human exposure. A variety of personnel are exposed to pyrethroids during manufacture and application, diet, and drinking water. Although these compounds are widely considered safe for humans [3], numerous studies have shown that very high exposure to pyrethroids might cause potential problems to man [4]. Such effects include suppressive effects on the immune system, endocrine disruption, lymph node and splenic damage, and carcinogenesis [5]. In addition, most synthetic pyrethroids possess apparent toxicity to fish and other aquatic organisms, including aquatic invertebrates, often at concentrations less than 1 μg L^-1 ^[6]. Therefore, it is important to develop a rapid and efficient disposal process to eliminate or minimize contamination of surface water, groundwater and agricultural products by pyrethroid insecticides.

Bioremediation strategies have potential for eliminating or reducing these residues. One such strategy would use enzymes to degrade the pesticide residues, for example, in bioreactors through which contaminated water could be passed, or in washing solutions after post-harvest disinfestation of fruit, vegetables or animal products to reduce residue levels and withholding times [1]. Suitable enzymes for degrading pesticide residues include hydrolases from bacteria, vertebrates and insecticide resistant insects [7-9].

Carboxylesterases represent a group of highly variable and multifunctional hydrolytic enzymes. They have potential for use in the hydrolysis and synthesis of important ester compounds of pharmaceutical, food, biochemical, and biological interests. Some pyrethroid carboxylesterases from pyrethroid-resistant insects have been purified and characterized [8]. Some pyrethroid-degrading bacteria including *Bacillus cereus *SM3 [9], *Pseudomonas Fluorescens *[10], *Vibrio hollisae*, *Burkholderia picketti*, and *Erwinia carotovora *have been isolated from soils and rivers [11], The only pyrethroid-degrading enzyme from *Bacillus cereus *SM3 was purified and characterized. These studies have indicated that the first step in the microbial degradation and detoxifiction of pyrethroid compounds is the hydrosis of carboxylester linkage, and demonstrated that these enzymes differ in substrate specificity, sensitivity to inhibitors, activation by metals and molecular mass. In a word, this method only focuses on the culturable portion of microorganisms. In fact, most microorganisms have not been researched due to limitations in culturing microorganisms, it is estimated that more than 99% of microorganisms are thought to be unculturable or difficult to culture in any given environment using standard cultivation methods and therefore not accessible as a source for finding useful biomolecules [12,13]. In order to search for new or improved bioactive products, the development of metagenomic technologies over the past few years has provided access to much of the prokaryotic genetic information available in environmental samples, independent of culturability [14]. Through metagenomic cloning, some genes encoding metagenomic lipases and esterases have been identified in metagenomic libraries from different environmental samples such as soil, water, thermal environment and deep-sea hypersaline anoxic basin [15-18].

In this report, we constructed a metagenomic library from vegetable soil for the isolation by functional expression screening of plasmid clones with esterase activity. Several clones with esterase activity were detected, and a novel pyrethroid-hydrolyzing esterase with higher activity and broader substrate specificities was selected for further characterization including thermal stability, optimal temperature and substrate specificity. To our knowledge, this is the first report so far on information about pyrethroid-hydrolyzing esterase gene from the unculturable bacterial genome. Further study is helpful to obtain excellent detoxifying enzyme for use as bioremediation agents.

## Results

### Construction of a metagenomic library and screening of a novel esterase gene

The prokaryotic DNA for metagenomic library, which was used in all experiments, was extracted from vegetable soil. A novel soluble esterase of Pye3 on an activity based strategy was designed. To test the quality of the library, 100 clones were randomly selected, and the recombinant plasmids were prepared. The average insert size was 4.2 kb, and sizes ranged from 3 to 8 kb. Out of approximately 93,000 colonies, 6 clones were identified by their bright blue color. The metagenomic library represented about 390 Mb of soil microbial community DNA. From these clones, the plasmids were isolated, purified and transformed to fresh *E. coli *TOP10 cells, all colonies turned blue again and the hydrolysis of pyrethroid was further tested. The ability of the only one positive blue colonies *E. coli *TOP10 (pZP6) to hydrolyze pyrethroid was confirmed by GC-MS analysis. Therefore, pZP6 was subjected to further analysis. The size of cloned fragment in plasmid pZP6 was reduced when the fragment was digested with various restriction enzymes, and the deletion derivatives were screened according to pyrethroid-hydrolyzing esterase activity, resulting in the subclone *E. coli *TOP10 (pZP6-3). The sequence analysis indicated that the pyrethroid-hydrolyzing esterase activity was attributed to a 2.6-kb *Eco*RI fragment. The sequence analysis of the insert DNA showed the presence of one open reading frame (ORF) 819 bp, encoding a polypeptide of 272 amino acids with a predicted molecular mass (Mr) of 31.15 kDa.

### Sequence analysis of pyrethroid-hydrolyzing esterase

**Figure 1:**
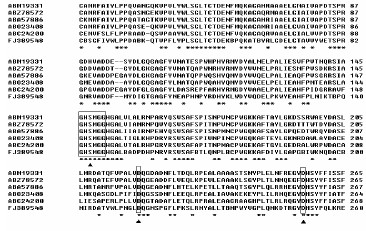
**Multiple alignment of the partial Amino acid sequences containing the conserved motifs of GxSxG and putative catalytic triad resides of esterases**. Except for Pye3, other protein sequences were retrieved from GenBank under following accession numbers: our metagenome (FJ389548), *Marinobacter aquaeolei *VT8 (ABM19331), *Shewanella halifaxensis *HAW-EB4 (ABZ78572), *Nitrosococcus oceani *ATCC 19707 (ABA57886), *Shewanella loihica *PV-4 (ABO23408), *Rhodospirillum rubrum *ATCC 11170 (ABC24200). The alignment was carried out using the Clustal W method. The open boxes indicate amino acid resides belonging to the putative catalytic triad resides, triangles denote the active site. The same amino acid resides are marked by (*).

**Figure 2:**
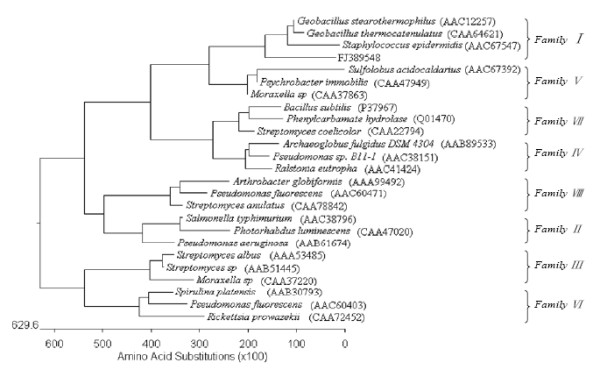
**Phylogenetic relationship of Pye3 and lipase/esterase proteins of 8 different families was performed using the program MEGALIGN (DNASTAR, Madison, WI)**. Except for Pye3, the other protein sequences for previously identified families of bacterial lipolytic and esterolytic enzymes were retrieved from GenBank under following accession numbers: our metagenome (FJ389548), *Pseudomonas fluorescens *(AAC60403), *Spirulina platensis *(AAB30793), *Rickettsia prowazekii *(CAA72452), *Arthrobacter oxydans *(Q01470), *Bacillus subtilis *(P37967),*Streptomyces coelicolor *(CAA22794), *Archaeoglobus fulgidus *DSM 4304 (AAB89533), *Pseudomonas *sp. B11-1 (AAC38151), *Ralstonia eutropha *(AAC41424), *Pseudomonas fluorescens *(AAC60471), *Streptomyces anulatus *(CAA78842), *Arthrobacter globiformis *(AAA99492), *Salmonella typhimurium *(AAC38796), *Photorhabdus luminescens *(CAA47020), *Pseudomonas aeruginosa *(AAB61674),*Moraxella *sp. (CAA37220), *Streptomyces albus *(AAA53485), *Streptomyces *sp. (AAB51445), *Geobacillus stearothermophilus *(AAC12257), *Geobacillus thermocatenulatus *(CAA64621), *Staphylococcus epidermidis *(AAC67547), *Moraxella *sp. (CAA37863), *Psychrobacter immobilis *(CAA47949), *Sulfolobus acidocaldarius *(AAC67392). The units at the bottom of the tree indicate the number of substitution events.

A blast search in the databases of NCBI revealed that the Pye showed moderate identity (45–49%) at the amino acid level with several carboxylesterases from *Shewanella loihica *PV-4 (132/266, 49%), *Marinobacter aquaeolei *VT8 (128/267, 47%), *Rhodospirillum rubrum *ATCC 11170 (130/263, 49%) and *Nitrosococcus oceani *ATCC 19707 (124/270, 45%). Multiple alignments of the deduced amino acids of Pye with the most homologous proteins were presented in Figure 1. The putative protein contained the conserved active site motif of the pentapeptide GxSxG found in most bacterial and eucaryotic serine hydrolases (residues from141 to 146)with a serine acting as the catalytic nucleophile, a conserved aspartate or glutamate and a histidine, together constituting a catalytic triad sequence (Ser 143, Asp214, His 251) [19,20]. An unrooted phylogenetic tree based on the amino acid sequences was constructed in order to further verify the evolutionary relationship of the Pye3 protein to other known lipase/esterase proteins, and 24 bacterial lipase/esterase proteins representing 8 different families were selected for the phylogenetic tree analysis [21]. As shown in Figure 2, the Pye3 protein belongs to Family I.

### Overexpression and purification of recombinant Pye3

**Figure 3:**
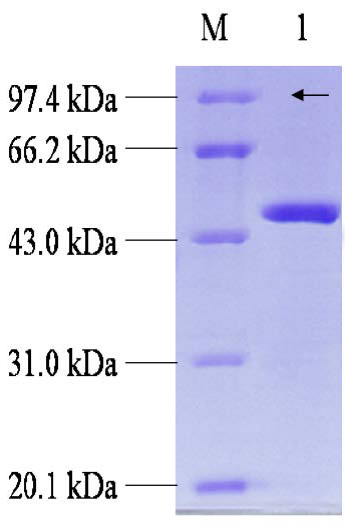
**SDS-PAGE analysis of the purified His_6_-tagged mature Pye3**. Arrowhead indicates target protein (lane 1), protein markers (lane M) stained with Coomassie blue. Markers from top to bottom are phosphorylase b (97.4 kDa), bovine serum albumin (66.2 kDa), ovalbumin (43 kDa), carbonic anhydrase (31 kDa) and trypsin inhibitor (20.1 kDa).

To investigate the biochemical property of Pye3, the cloned gene was subcloned in frame with a six-histidine tag sequence into a T7 RNA polymerase derived *E. coli *expression vector of pET 32a + (Novagen) and expressed in *E. coli *BL21 (DE3) with 0.5 mM IPTG induction. The recombinant Pye3 was purified by Ni-NTA chromatography. Taking into consideration that the resulting recombinant protein should consist of the 272 amino acids with an N-terminal fusion of 156 amino acids corresponding to thioredoxin tag (Trx-tag), polyhistidine tag (His-tag), S. tag epitope (S. tag) and a unique thrombin cleavage site (Thrombin), and its total molecular mass should be about 48 kDa. It was observed about 48 kDa in a Coomassie-stained SDS-PAGE (Fig. 3). This result is in agreement with the calculated molecular mass of the predicted amino acid sequence (31,15 kDa). The relative molecular mass of native enzyme estimated by gel filtration on a calibrated column of Sephacryl 200 HR was 73,000 Da. Hence, it is assumed that the purified enzyme (Pye3) is a monomer. The pI value was estimated to be 5.2.

### The effect of pH and temperature on catalytic activity and stability

With ρ-nitrophenyl acetate as a substrate, the pH ranged from 3.5 to 10.0 and the temperature ranged from 20 to 70°C. The absorption of ρ-nitrophenol varies when pH is altered because of changes in equilibrium between ρ-nitrophenol and ρ-nitrophenoxide. Therefore, the release of ρ-nitrophenol was monitored at 384 nm. The pH-activity profile of the enzyme was bell-shaped, with maximum values at pH 7.0. The enzyme was found to be stable in the pH range of 5.5 to 9.0. The optimal temperature for the enzyme was 40°C. The enzyme was fairly stable at up to 45°C and had 54% of its activity at 50°C. It was completely inactivated at 65°C.

### The effects of reagents and metal ions on enzyme activity

The presence of Hg^2+ ^and Ag^+ ^caused a complete inhibition at 0.5 mM, while less pronounced effect was observed in the presence of the divalent cations (0.5 mM). The enzyme activity was strongly inhibited by 0.1 mM ρ-chloromercuribenzoate, whereas the chelating agent EDTA and phenanthroline (1 mM) showed little effect on the enzymatic activity.

### Substrate specificity

The substrate specificity towards ρ-nitrophenyl esters of various fatty acids was shown in Table 1. Pye3 showed the highest activity with ρ-nitrophenyl caproate (426 U/mg) among the ρ-nitrophenyl esters examined. Both *K*_m _and *k*_cat _values of purified Pye3 decreased with increases in aliphatic chain length up to C_4_. The comparison of catalytic efficiency values (*k*_cat_/*K*_m_) for various substrates indicated that these values were dependent on the aliphatic chain length of substrate. Short-chain ρ-nitrophenyl esters seemed to be preferred substrates, whereas ρ-nitrophenyl esters of longer-chain fatty acids were poor substrates, Pye3 had no activity against ρ-nitrophenyl myristate and ρ-nitrophenyl palmitate. Activity to ρ-nitrophenyl esters of shorter-chain fatty acids was lower than that of ρ-nitrophenyl butyrate. Taking into consideration that lipases prefer substrates with relatively long aliphatic chains, these results showed the purified enzyme (Pye3) is an esterase and not a lipase.

A range of pesticides such as organophosphorus insecticide malathion whose structures are similar to pyrethroid, *cis*-permethrin, *trans*-permethrin, cypermethrin, fenvalerate, and deltamethrin were tested for substrate specificity of the recombinant Pye3. The Pye3 hydrolyzed the pesticides tested at different hydrolysis rate (Table 2), the *trans*-permethrin was hydrolyzed most rapidly, while deltamethrin was the least readily attacked, *cis*-permethrin was hydrolyzed approximately equal rate toward *trans*-permethrin. The *K*_m _and *k*_cat _values were calculated by fitting the data to Michaelis-Menten equation (Table 2). The purified enzyme showed comparable affinity against a range of structurally similar pesticides with *K*_m _values ranging 0.1–1.41 μM.

## Discussion

Soil is often considered to be one of the main reservoirs of microbial diversity on the planet [22]. However, the inability to cultivate most soil bacteria hampered fundamental attempts to determine the diversity of the prokaryotic world and limited its industrial exploitation. In the last 20 years, new methods have been developed to overcome these limitations based on the direct extraction of DNA from bacteria in their natural environment. Metagenomic approaches promise the accessibility of the genetic resources and their potential biotechnological applications [13,23-25].

In fact, like most other proteins screened from metagenomic libraries, no information about a source microorganism could be obtained. Therefore, the success rate (the number of positives divided by the library size) is relatively low [17,26]. In this study, the metagenomic library represented about 390 Mb of soil microbial community DNA was constructed. Through screening 93,000 clones, 6 esterase-positive transformants displayed blue color in LB containing X-caprylate. Of which only one positive clone among them was able to hydrolyze pyrethroid by pyrethroid-hydrolyzing experiment. This confirms the general trend that the discovery of novel pyrethroid-hydrolyzing enzyme from the metagenome is not straightforward. The reason for low yield of novel pyrethroid-hydrolyzing esterases can be attributed to a number of different factors, including the library construction (source of DNA, number of clones, and average insert size of metagenomic DNA), the probable unrecognition of regulatory elements from unknown bacteria in *E. coli*, difficulties of expression in heterologous host, the formation of inclusion bodies, toxicity of expressed protein to host, sufficient quantities and stability of genes encoding proteins and the choice of host organism. Therefore, this suggests that the discovery of specific genes in a complex metagenome is technically challenging.

Fortunately, metagenomic libraries were constructed by using the vector pZErO-2, and a novel pyrethroid-hydrolyzing esterase gene was thus isolated and characterized. Taking into consideration the complexity in the course of directly cloning of pyrethroid-hydrolyzing esterase gene, the ability to produce a blue precipitate pertinent to esterase enables screening for novel esterases from metagenomic libraries using *E. coli *as a host organism. In this study, we initially selected 6 blue color transformants with the esterase activity in LB agar plates containing 100 μM X-caprylate, the ability of those blue transformants with the esterase activity to hydrolyze permethrin was subsequently demonstrated by assaying permethrin reduction, and further analysis determined that only one transformant with the esterase activity hydrolyzed permethrin. These results showed that not all esterases are capable of degrading pyrethroids. Furthermore, the Pye3 not only efficiently hydrolyzed ρ-nitrophenyl esters of medium-short chain fatty acids, but also degraded pyrethroid pesticides tested, indicating that the Pye3 is an esterase with broader specificity. Although there have been some reports on pyrethroid hydrolase from pyrethroid-resistant insects, mammal organ, *Aspergillus niger *and *Bacillus cereus *[1,3,9,27], some nucleotide sequences of the pyrethroid-hydrolyzing enzyme genes were available (accession no. NM-133960, accession no. NM-144930, accession no. AY487948, accession no. Q964Q7, accession no. Q27698) [28,29], the pyrethroid pesticide degradative gene from the metagenomic library has not been previously reported. To our knowledge, this is the first one to be determined for pyrethroid-hydrolyzing enzyme gene from the metagenomic library constructed from vegetable soil.

The gene (*pye*) was cloned and expressed in *Escherichia coli *BL21 (DE3), and its product was purified to apparent homogeneity and characterized. As a monomeric 31.15 kDa protein, the molecular mass of the Pye3 is smaller than those of the purified permethrinase (61,000 Da) from bacteria, the pyrethroid hydrolase (56,000 Da) from Aspergillus niger ZD11, pyrethroid-hydrolyzing carboxylesterase (60,000 Da) from mouse liver microsomes, and carboxylesterase E3 (58,600 Da) from *Nephotettix cincticeps *Uhler. The pH optimum of the Pye3 (pH 7.0) was lower than that recorded from *Bacillus cereus *(pH 7.5) and higher than that reported from *Aspergillus niger *ZD11 (pH 6.5). The optimal temperature of 40°C is similar to that recorded from *Bacillus cereus *(37°C) and lower that reported from *Aspergillus niger *ZD11 (45°C) [3,9].

The substrate specificity on different substrates was studied with the purified recombinant enzyme. The striking feature for ρ-nitrophenyl caproate and ρ-nitrophenyl caprylate was quite different from the specificities of other microbial esterases [30], which are mainly specific for ρ-nitrophenyl esters of short-chain fatty acids, only the enzymes from *Bacillus. stearothermophilus *[31], *Sulfolobus acidocaldarius *[32], *Bacillus licheniformis *[33], and *Lactobacillus casei *CL96 [34] showed specificity patterns are similar to that of Pye3. Since the purified recombinant enzyme hydrolyzed cypermethrin, permethrin, fenvalerate, deltamethrin, and malathion. Therefore, pyrethroid-hydrolyzing esterase seems to be capable of hydrolyzing a relatively wide range of compounds with similar chemical linkage, suggesting that it possessed broader substrate specificities, and this feature is same as pyrethroid hydrolase from *Aspergillus niger *ZD11 [3]. However, this observation does not quite agree with data reported by Motoyama et al. and Stok et al. [27]. The pyrethroid-hydrolyzing carboxylesterase (BAC36707) from mouse liver microsomes did not hydrolyze malathion. On the other hand, Motoyama et al. found that one of five forms of carboxylesterases degraded malathion twice as fast as the fenvalerate, and three other forms possessed approximately equal activity toward these two insecticides. In a previous paper, there was a preference in both mammals and insects carboxylesterases for permethrin to cypermethrin, and *trans*-permethrin to *cis*-permethrin [8,27], while carboxylesterase from *Nephotettix cincticeps *Uhler prefered *cis*-permethrin to *trans*-permethrin. But the pyrethroid-hydrolyzing esterase possessed approximately equal activity toward permethrin isomers. Therefore, it lacked stereoselectivity, and this feature is different from that of carboxylesterase E3 from *Nephotettix cincticeps *Uhler [8] and carboxylesterase (BAC36707) [27]. In addition, the apparent *K*_m _values obtained for the purified enzyme (Pye3) were lower than those for pyrethroid-hydrolyzing carboxylesterase (BAC36707) from mouse liver microsomes, carboxylesterase E3 from *Nephotettix cincticeps *Uhler and pyrethroid hydrolase from *Aspergillus niger *ZD11 when the same substrates were used, indicating a higher affinity for these substrates in the case of the Pye3. The comparison of *K*_m _and *k*_cat _revealed that he pyrethroid-hydrolyzing esterase has about 14-fold higher affinity towards *trans*-permethrin than deltamethrin and can hydrolyze the former about 173-fold faster than the latter. The catalytic efficiencies (*k*_cat_/*K*_m_) are considered as a measurement of the enzyme,s specificity, among these substrates indicate that *trans*-permethrin is clearly the preferred substrate.

## Conclusion

In summary, the pyrethroid-hydrolyzing esterase gene was successfully cloned using metagenomic DNA combined with activity-based functional screening from soil, the recombinant Pye has been purified and characterized. Taking into consideration that pesticidal residues resulting from agricultural production are complex mixtures, enzymatic bioremediation requiring the development of specific enzymes for each compound or isomer is unrealistic, therefore, the broader substrate specificities and higher activity of the pyrethroid-hydrolyzing esterase were necessary to fulfill the practical requirements of bioremediation to enable its use in situ for detoxification of pyrethroids where they cause environmental contamination problems. Further studies will supply important data for future application of novel pyrethroid-hydrolyzing esterase for promising environmental protection.

## Methods

### Chemicals and Reagents

Cypermethrin (98%), *trans*-Permethrin and *cis*-Permethrin (99%), Fenvalerate (98%), malathion (98%), and Deltamethrin (98%) were kindly provided by Zhong shan Pesticide Factory (Guang dong, China). All ρ-nitrophenyl esters were purchased from Sigma. All other chemicals and reagents were of analytical grade and were purchased from commercial sources, unless otherwise stated.

### Strains, media, and plasmids

*E. coli *TOP10 was used as a host for recombinant plasmids. The pET-32a (+) (Novagen) was used as an overexpression vector to produce the target protein.*E. coli *BL21 (DE3) was used as a host for expression of the *pye3 *gene under the control of the T7 promoter. *E. coli *transformants were grown at 37°C in Luria-Bertani (LB) broth containing, when necessary, the LB medium was supplemented 50 μg/mL kanamycin, unless otherwise stated.

### DNA manipulation

Routine DNA manipulations were carried out according to standard techniques. Restriction enzymes and DNA polymerase were purchased from Takara (Dalian, China). Each enzyme was used according to the recommendations of the manufacturer. DNA ligations were performed using T4 DNA ligase (Fermentas). Plasmids were prepared from *E. coli *by using a QIAGEN miniplasmid purification kit according to the manufacturer's instructions (QIAGEN Inc.). DNA fragments were isolated from agarose gels by using a QIAquick gel extraction kit (QIAGEN Inc.). The *E. coli *TOP10 and vector pZErO-2 were purchased from Invitrogen (Invitrogen, USA). Electroporation was performed with a Gene-Pulser II electroporation apparatus (Bio-Rad Laboratories).

### Sequencing and analysis of pyrethroid-hydrolyzing esterase gene

Sequencing reactions were performed using a BigDye sequencing kit according to the instructions of the manufacturer. DNA sequencing of positive plasmid clone (pZP6-3) was analyzed on ABI 377 DNA sequencer. Sequence manipulation, open reading frame (ORF) searches, and multiple alignments among similar enzymes were conducted with DNASTAR software. Database homology search was performed with BLAST program provided by NCBI. The conserved patterns of discrete amino acid sequences related enzymes were analyzed by Clustal W program.

### DNA extraction from soil samples

The topsoil samples (5 to 10 cm) from vegetable soil were used for the experiments. Samples were collected and stored at -80°C until the DNA extraction was performed. Extraction of the total genomic DNA from vegetable soil was performed using with the Fast DNA^® ^SPIN kit for soil according to the recommendations of suppliers (MP Biomedicals, USA).

### Construction of genomic libraries and screening for pyrethroid-hydrolyzing esterase gene

The metagenomic library was constructed from environmental DNA isolated from vegetable soil using protocols provided by the manufacturer. DNA fragments (3 to 8 kb) obtained after partial *Sau3*AI digestion were ligated into the *Bam*HI restriction site of the pZErO-2 vector, which had been previously digested with *Bam*HI. *E. coli *TOP10 was transformed by electroporation with the library and plated onto Luria-Bertani (LB) agar plates containing 50 μg/mL kanamycin, 100 μM 5-bromo-4-chloro-3-indolylcaprylate (X-caprylate) and 0.5 mM isopropyl-β-D-thiogalactopyranoside (IPTG). A total of approximate 93,000 transformants were generated. A functional esterase screening was visualized performed by blue color, which was resulted from the hydrolysis of X-caprylate. To avoid the isolation of false-positive clones, plasmid DNA was isolated from the positive clones obtained in the initial screening and retransformed, and the new clones were examined on the same type of indicator plates for esterase activity. The 6 blue colonies were further tested for its ability to hydrolyze permethrin through (GC-MS) analyses [3]. The only one transformant with pyrethroid-hydrolyzing esterase was obtained and confirmed. Subsequently, the plasmid was subjected to restriction analysis with *Bam*HI to identify the uniqueness of the clone. Unique clones were sequenced to identify the inserted fragment of metagenomic DNA. The fragment size of the plasmids was 6.3 kb. The size of the insert DNA fragment of the plasmid pZP6 was reduced by using the *Eco*RI restriction site. The resulting DNA fragment of 2.6 kb from plasmid pZP6 was ligated into a *Eco*RI -digested pZErO-2 plasmid. The ligated DNA was introduced into *E. coli *TOP10, the resulting clone *Escherichia coli *TOP10 (pZP6-3) was tested for its ability to hydrolyze permethrin through (GC-MS) analyses [3].

### Cloning and over-expression and purification of pyrethroid-hydrolyzing esterase

The putative esterase gene was amplified from the pZP6-3 plasmid by using the primers and to introduce *Bam*HI and *Hind*III restriction sites for cloning in the pET32a (+). The following primers were used: *pye*-fw (5-CGC***GGATCC***ATGCCAAGTGATCAAAGAG; the *Bam*HI site is shown in italics) and *pye*-rv (5-CCC***AAGCTT***GTTGGTGTCCTGCAGGAAAC; the *Hind*III site is shown in italics). The PCR product was digested with *Bam*HI/*Hind*III, and then ligated into *Bam*HI/*Hind*III digested expression vector pET-32a (+), and transformed into *E. coli *BL21 (DE3) (Stratagene). The E. coli transformed with this plasmid was plated on LB agar containing 50 μg/mL kanamycin. The transformant was grown in a 250-ml flask containing 50 ml LB medium supplemented with 50 μg/mL kanamycin at 37°C until the cell concentration reached OD_600 _nm of 0.6, and 0.1 mM isopropyl-β-D-thiogalactopyranoside (IPTG) of a final concentration to induce target protein expression. After incubation at 25°C for 18 h with shaking at 200 rpm, Cells were harvested by centrifugation (6, 000 *g *for 20 min at 4°C) and resuspended in 50 mM Tris-HCl (pH 7.6)-10 mM EDTA and disrupted by sonication for 10 s in ice-water bath. The cell debris was removed by centrifugation at 10,000 *g *for 5 min at 4°C. The clear supernatant was collected and the recombinant Pye was purified with his-tag affinity column. The supernatant was applied to a Ni-nitrilotriacetic acid (Ni-NTA) affinity chromatography column (Qiagen, Hilden, Germany), equilibrated with buffer A (10 mM Tris-HCl, 500 mM NaCl, 5 mM imidazole, pH 8) at a flow rate of 0.5 mL/min). The bound protein was eluted with buffer B (10 mM Tris-HCl, 500 mM NaCl, 500 mM imidazole, pH 8) at 4°C. The purity of the enzyme was estimated by SDS (sodium dodecyl sulfate)-PAGE (polyacrylamide gel electrophoresis) in the eluted fractions, using 12% polyacrylamide running gels. Protein concentration was determined by the method of Bradford, bovine serum albumin (Sigma) was used as standard for calibration [35]. Enzyme samples were stored at -20°C until further use.

### Determination of molecular mass and isoelectric point

The molecular mass of the denatured protein was determined by sodium dodecyl sulfate-polyacrylamide gel electrophoresis (SDS-PAGE). 12% SDS-PAGE was prepared by the method of Laemmli [36]. Proteins were stained with Coomassie brilliant blue G. The molecular mass of the enzyme subunit was estimated using protein marker as standards. The molecular mass of the native protein by gel filtration on a Superose 12HR 5/30 column, gamma globulin (160, 000 Da), bovine serum albumin (67, 000 Da), ovalbumin (43, 000 Da), carbonic anhydrase (30, 000 Da), was used as the reference proteins. Isoelectric point (pI) was estimated by PAGE with 6.25% Ampholine (pH 3.5~10) in a gel rod (0.5 by 10 cm) using a kit for Isoelecric Focusing Calibration according to recommendations by the supplier.

### Analysis of enzyme activity

The esterase activity against ρ-nitrophenyl esters was determined by measuring the amount of ρ-nitrophenol released by esterase-catalyzed hydrolysis. The hydrolysis of substrate was performed at 30°C for 10 min in 50 mM sodium phosphate buffer (pH 7.0) containing 1% acetonitrile. The production of ρ-nitrophenol was monitored at 405 nm by Labsystems Dragon Wellscan MK3. One unit of enzyme activity was defined as the amount of enzyme that produced 1 μmol of ρ-nitrophenol per minute from substrate under these conditions [3,17,37]. The pyrethroid-hydrolyzing esterase activities against pesticides and the pesticide assay were performed as described by Liang et al. [3].

The optimum pH of the pyrethroid-hydrolyzing esterase was measured using ρ-nitrophenyl caproate as a substrate at 30°C. The buffers (at a final concentration of 50 mM) used for the measurement were as below: citric acid-NaOH (pH 3.5 to 5.5); potassium phosphate (pH 5.0 to 7.0); Tris-HCl buffer (pH 6.5 to 9.0), glycine-NaOH buffer (pH 8.5 to 10.0). Overlapping pH values were used to verify that there were no buffer effects on substrate hydrolysis. The optimum temperature was determined analogously by measuring esterase activity at pH 7.0 in the temperature range of 20–70°C. The pH stability was tested after incubation of the purified enzyme for 2 h at 30°C in the above different buffers. Temperature stability was measured after incubation of the purified enzyme in 50 mM sodium phosphate buffer (pH 7.0) for 1 h at different temperature [17,38]. The effects of various chemicals (CaCl_2_, MgSO_4_, CuSO_4_, ZnSO_4_, MnCl_2_, AgNO_3_, HgCl_2_, EDTA, phenanthroline, ρ-chloromercuribenzoate) on the pyrethroid-hydrolyzing esterase activity were investigated by addition of the tested compounds into the reaction mixture, which was preincubated for 30 min at 30°C. The activity was then measured described above and expressed as a percentage of the activity obtained in the absence of the added compound. Taking into consideration the fact that the poor solubility of most metal ions in phosphate buffer, we use 50 mM N- (2-hydroxyethyl)-piperazine-N'- (2-ethanesulfonic acid)-NaOH buffer (pH 7.0) in place of 50 mM sodium phosphate buffer.

### Determination of kinetic parameters

Kinetic parameters against ρ-nitrophenyl esters were determined by measuring enzyme activity using ρ-nitrophenyl acetate ranging from 0.02 to 1.5 mM, ρ-nitrophenyl propionate ranging from 0.01 to 0.8 mM, ρ-nitrophenyl butyrate ranging from 0.008 to 0.5 mM, ρ-nitrophenyl caproate ranging from 0.003 to 0.2 mM, ρ-nitrophenyl caprylate ranging from 0.005 to 0.4 mM, ρ-nitrophenyl laurate and ρ-nitrophenyl myristate ranging from 0.03 to 2 mM as a substrates in 50 mM sodium phosphate buffer (pH 7.0) containing 1% acetonitrile at 30°C. An additional 0.04% Triton X-100 was included in the reaction mixture in the case ρ-nitrophenyl caprylate. With ρ-nitrophenyl palmitate as a substrate, 4% 2-propanol was included in the reaction mixture in order to solubilize the substrate. Kinetic parameters against different pesticides were analogously determined by measuring enzyme activity over a range of final concentrations from 0.005 to 7 μM dependent on different pesticides. All initial velocities were determined at 5 time points which no more than 10% of the substrate had been consumed, and solution content never exceeded 1% of the total assay volume, so the decrease in substrate concentration remains linear with time over the period of measurement and the rate was almost constant throughout the assay, r-square values ranged from 0.963–0.971 according to different substrates. Initial reaction velocities measured at various concentrations of substrates were fitted to the Lineweaver-Burk transformation of Michaelis-Menten equation [39]. Kinetic analyses by curve fitting were performed with the SigmaPlot software [3].

### Nucleotide sequence accession number

The nucleotide sequence data reported here have been submitted to the nucleotide sequence databases under accession number (FJ389548).

## Competing interests

The authors declare that they have no competing interests.

## Authors' contributions

GL: he carried out gene cloning, expression and characterization of protein together with KW. KW: he constructed metagenomic library from soil, carried out the bioassay of enzyme activity and analyzed data. YHL: he conceived the study, designed and supervised the experiments, and drafted and revised the manuscript. All authors have read and approved the manuscript.
